# Study of a Novel Gemini Viscoelastic Surfactant with High Performance in Clean Fracturing Fluid Application

**DOI:** 10.3390/polym10111215

**Published:** 2018-11-01

**Authors:** Wenlong Zhang, Jincheng Mao, Xiaojiang Yang, Heng Zhang, Zhaoyang Zhang, Bo Yang, Yang Zhang, Jinzhou Zhao

**Affiliations:** State Key Laboratory of Oil and Gas Reservoir Geology and Exploitation, Southwest Petroleum University, Chengdu 610500, China; 201711000107@stu.swpu.edu.cn (W.Z.); 201811000155@stu.swpu.edu.cn (H.Z.); 201611000102@stu.swpu.edu.cn (Z.Z.); 201611000112@stu.swpu.edu.cn (B.Y.); 201811000149@stu.swpu.edu.cn (Y.Z.); zhaojz@swpu.edu.cn (J.Z.)

**Keywords:** Gemini cationic surfactant, salt-induced, wormlike micelles, viscoelasticity, clean fracturing fluid

## Abstract

Gemini surfactant, as a functionally flexible polymer-like material in the aqueous solution, has attracted increased attention in reservoir stimulation of hydraulic fracturing in recent decades. A new Gemini cationic viscoelastic surfactant named JS-N-JS, which has a secondary amine spacer group and two ultra-long hydrophobic tails, was synthesized from erucamidopropyl dimethylamine, diethanolamine, and thionyl chloride as a thickener for hydraulic fracturing fluid. Compared with some Gemini cationic surfactant with methylene spacer, JS-N-JS showed a lower critical micellar concentration (CMC) and higher surface activity due to the hydrogen bond formed between the secondary amine and water molecule intends to reduce electrostatic repulsion, which is more beneficial to be the fracturing fluid thickener. Moreover, the performance of JS-N-JS solution can be further improved by salts of potassium chloride (KCl) or sodium salicylate (NaSal), while organic salt behaved better according to the measurements. The SEM observation confirmed that JS-N-JS/NaSal system owned a tighter network microstructure, and JS-N-JS/NaSal system exhibited a distinct superior viscoelasticity system at a sweep frequency of 0.1–10 Hz. As a fracturing fluid, the solution with a formula of 30 mmol JS-N-JS and 100 mmol NaSal was evaluated according to the petroleum industrial standard and presented excellent viscoelastic properties, the viscosity of which can maintain above 70 mPa·s for 110 min under a shear rate of 170 s^−1^ at 120 °C. Meanwhile, the drag reducing rate of the formula could reach above 70% with the increase of shear rate. Finally, the viscous fracturing fluid can be broken into the water-like fluid in 1.2 h after being fully exposed to hydrocarbons and the water-like fluid presented a low damage to the tight sand reservoirs according to the core flooding experiments, in which the permeability recovery rate can reach 85.05%. These results fully demonstrate that the JS-N-JS solution fully meets the requirement of the industrial application of hydraulic fracturing.

## 1. Introduction

As a functionally flexible polymer-like material in the aqueous solution, viscoelastic surfactant (VES) has great potential to be a thickener for the fracturing fluid, and it has attracted an increasing attention in recent decades due to its polymer-like behaviors in aqueous solution and stimuli-response properties [1,2,3]. Different from the normal polymer applied in the hydraulic fracturing fluid, VES can self-assemble into long and flexible wormlike micelles in the aqueous solution, behaving as a crosslinked polymer. Stimulus conditions [3,4,5,6,7] such as light, pH, temperature, and electrolyte can induce various responses of VES molecule aggregation, thus the macroscopic nature of viscoelasticity can be adjusted by changing the stimulus conditions [8]. Due to the “magical” properties of the viscoelastic surfactants, they have been welcomed in many industrial applications such as smart optical systems [9], drug delivery [10], template synthesis [11], biosensors [12] in past decades. Recently, they are introduced into the petroleum industry, applied to enhanced drag reduction [13,14,15], reservoir stimulation [16,17,18,19,20] and oil recovery [21,22].

**Scheme 1:**
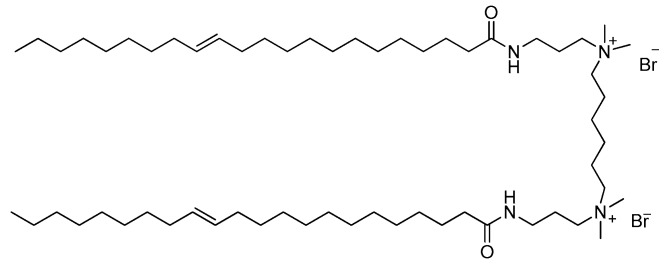
Structure of the Gemini cationic surfactant C25-6-C25 [39].

The hydraulic fracturing technique as the most common reservoir stimulation treatment has been applied in low-permeability reservoir stimulation for several decades, for which the fracturing fluid must own viscosity and elasticity properties to create an artificial fracture and transport proppant into the cracks [23,24,25]. Soluble macromolecular polymers such as guar gum and acrylamide polymers have been widely used as thickeners for fracturing fluid thickener in past decades [26]. The crosslinked polymer solution owns good viscoelastic properties, which can effectively transport proppant into the artificial cracks. However, the insoluble residue of guar gum or acrylamide polymers causes serious formation damage in the form of plugging the pore throat. In addition, the incomplete gel breaking results in detrimental effects on reservoir stimulation [27]. Moreover, the preparation of the guar gum or polymer fracturing fluid system is very cumbersome due to the complicated essential additives such as crosslinker, clay stabilizer, gel breaker and bactericide [26]. Thus, fracturing fluids with low damage (clean fracturing fluid) draw more and more attention in low-permeability or tight reservoir development. Fortunately, the wormlike micelles assembled by low molecular weight VES and the entangled networks impart very nice viscoelastic properties to the aqueous solution of VES, making the solution present analogous characteristics to polymer solutions [28,29]. Schlumberger firstly successfully applied a cationic viscoelastic surfactant for fracturing fluids in 1997 [19], called polymer-free fracturing fluid. The VES fracturing fluid showed many advantages, especially for the low-permeability reservoir, including low damage, low drag friction, free of cross-linker and biocides. Unlike polymers or guar gum gel, wormlike micelles collapse into spherical micelles or emulsions when exposed to hydrocarbon in the reservoir, which imparts the VES fracturing fluid residue free and easy to flow back [30,31]. The developed viscoelastic surfactants include anionic VES, cationic VES, and zwitterionic VES, among which cationic VES is applied most widely to the VES fracturing fluid [16,32,33,34]. Most of these viscoelastic surfactants were single-chain surfactants and deficiencies have limited their further application in extreme reservoir conditions. The traditional single-tailed surfactants possess high critical micelle concentrations (CMC) and poor surface activities, and the VES fracturing fluid prepared from the single tailed surfactants exhibit poor temperature and shear resistance [8,35,36]. Gemini surfactant as a superior type of surfactant was firstly reported in 1971, while not applied in oilfield [37]. The special structure of Gemini surfactant is made up of two single-chain surfactants that linked by a spacer group. The structure of the spacer group greatly affects the properties of the Gemini surfactant. Therefore, the performance of the Gemini surfactant can be improved by the modification of the spacer group [38]. On the other hand, the length of the hydrophobic chain is also crucial to the properties of the Gemini surfactant. The spacer group weakens the electrostatic repulsion between the head groups through strong chemical bonds. Therefore, the Gemini surfactants own superior properties compared to single-tailed surfactants, such as higher surface activity, lower CMC, contrasting self-assembly behavior and better rheological behaviors. In 2017, a Gemini surfactant of C25-6-C25 (Scheme 1) synthesized by Yang et al. exhibited good performance when applied to hydraulic fracturing fluid [39]. 

To further improve the performance, a method of the spacer modification was explored in this work. In addition, induction of organic salt (NaSal) and inorganic salt (KCl) was investigated to optimize the VES fracturing fluid formula. Finally, a VES fracturing fluid with the good performance was achieved.

## 2. Experimental

### 2.1. Materials

Diethanolamine, calcium chloride, ethanol, and acetone were purchased from KeLong Chemical Co., Ltd. (Chengdu, China). Erucamidopropyl dimethylamine was purchased from Shanghai Winson New Material Co., Ltd. (Shanghai, China). Thionyl chloride, potassium chloride, Sodium salicylate, and sodium carbonate were obtained from Shanghai Aladdin BioChem Technology Co., Ltd. (Shanghai, China). The chemicals, except erucamidopropyl dimethylamine (industrial grade), were chemical grade and without further purified. Deionized water was prepared in the lab and used in all tests.

### 2.2. Synthesis of the Gemini Cationic Surfactants

(1) Synthesis of the 2,2′-dichloro-diethylamine hydrochloride

**Scheme 2:**
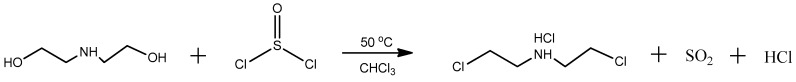
Synthesis of intermediate.

The intermediate, 2,2′-dichloro-diethylamine hydrochloride, was synthesized from thionyl chloride and diethanolamine. Diethanolamine (10.51 g, 100 mmol) was dissolved in chloroform (100 mL) and poured into a single-necked flask, and the single-necked flask was placed in an ice bath. Thionyl chloride (26.17 g, 220 mmol) was dissolved in chloroform (100 mL) and then added into the single-necked flask dropwise by constant pressure funnel. The SO_2_ and HCl generated by the reaction was imported into sodium hydroxide concentrated solution. After adding thionyl chloride, the single-necked flask was moved into an oil bath and stirred at 50 °C for 5 h. The synthetic route is shown in Scheme 2, and the generated gas continued to be imported into sodium hydroxide concentrated solution. The solution in the single-necked flask was cooled to 15 °C after 5 h, and then the product precipitated from the solution. Sucking filter was used to remove the solution and obtain the dry product.

(2) Synthesis of JS-N-JS

**Scheme 3:**
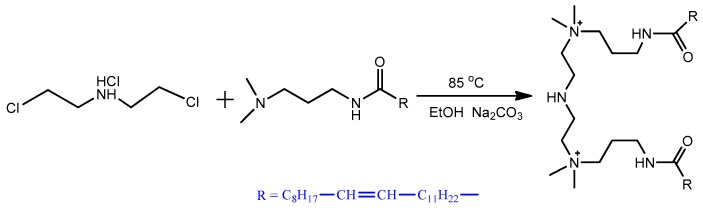
Synthesis of JS-N-JS.

The Gemini surfactant of JS-N-JS was synthesized from the intermediate (2,2′-dichloro-diethylamine hydrochloride) and erucamidopropyl dimethylamine. The erucamidopropyl dimethylamine (205 mmol, 86.72 g) and 2,2′-dichloro-diethylamine hydrochloride (100 mmol, 17.84 g) were added into a 500 mL single-necked flask (equipped with a condenser tube) and dissolved by 250 mL ethanol. The mixture was stirred at 85 °C for 24 h and the synthetic route is shown in Scheme 3. After 24 h, the sodium carbonate (100 mmol, 10.6 g) was added into the flask, and the mixture continued to be heated for 1 h to remove the HCl on the secondary amine. After cooling down, the solid phase in the mixture was removed by sucking filter, and the ethanol was removed by vacuum rotary evaporation and the product (JS-N-JS) was washed by acetone to remove the residual erucamidopropyl dimethylamine.

### 2.3. Experimental Tests

The molecular structures of JS-N-JS were characterized by a Nicolet 6700 FI-IR spectrometer (Nicolet, Madison, WI, USA) at ambient temperature, and the ^1^H NMR spectrums of the JS-N-JS was recorded in CDCl_3_ at 400 MHz by a Bruker AVANCE III HD 400 NMR spectrometer (Bruker, Karlsruhe, Germany) at ambient temperature.

A surface tension meter (Shanghai Hengping Instrument, Shanghai, China) was employed to determine the Critical Micelle Concentration (CMC) of JS-N-JS by surface tension measurement at 25.00 ± 0.05 °C. The measurement results were also used to study the variation of CMC value with the variation of added salt concentration. The apparent viscosity of the surfactant/salt solution was measured by NDJ-95A viscometer (Shanghai, China) with various concentrations. A HAAKE MAR III RS 600 Rheometer (Thermo Scientific, Munich, Germany) equipped with a high pressure sealed cell was employed to study rheological properties. Viscoelasticity was evaluated by Anton Paar physical MCR 301 Rotational Rheometer (Anton Paar, Graz, Austria). Microstructure analysis using a Cryo-environmental Scanning Electron Microscope (FEI, Hillsboro, OR, USA) was applied to help understand the macroscopic properties of the solution. The fluid samples were frozen at −165 °C to keep the microstructure intact, before scanning.

The thermo-shear resistance, static proppant suspension test, permeability recovery experiment, and gel breaking performance were evaluated by referring to the SY/T 6376-2008 [40] which is a recommended practice on measuring the properties of the water-based fracturing fluid.

## 3. Results and Discussion

### 3.1. Structural Characterization

**Figure 1:**
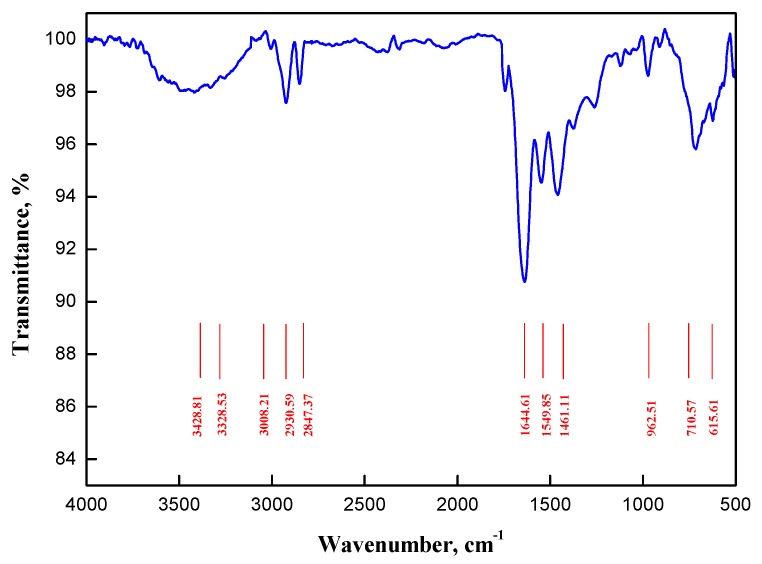
Infrared spectrum of JS-N-JS.

The structure of JS-N-JS was characterized and confirmed by FT-IR and ^1^HMR. Figure 1 shows the FT-IR spectrum of JS-N-JS.

The peaks at 3008.21, 2930.59, and 2847.37 cm^−1^ correspond to the stretch vibration absorption associated with C–H, –CH_3_, and –CH_2_–, respectively. The absorption peaks at 3428.81 and 1549.85 cm^−1^ are caused by the amide N–H stretching and bending vibrations. The absorption peak at 1644.61 cm^−1^ corresponds to the C=O stretching vibration. The peak at 3328.53 cm^−1^ corresponds to the stretch vibration absorption of the secondary amide N–H, while the peaks at 962.51, 710.57, and 615.61 cm^−1^ correspond to the bending vibration absorption of C–H, –CH3, and –CH2–, respectively.

**Figure 2:**
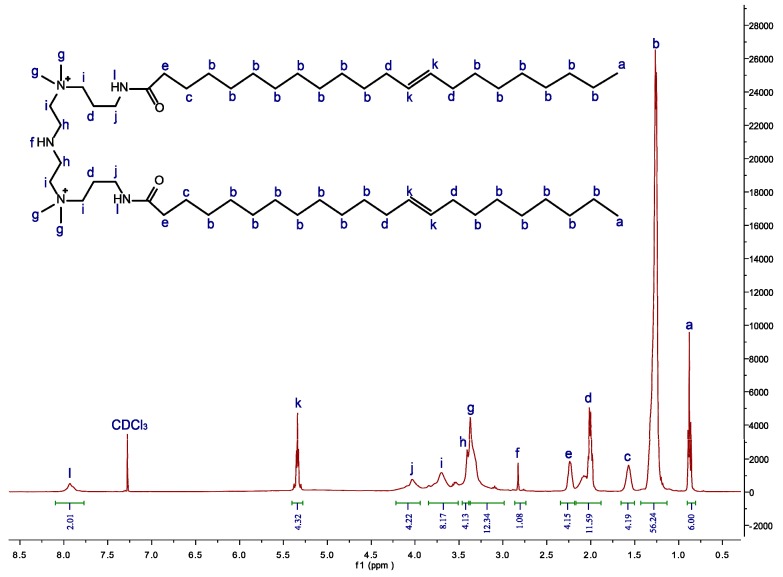
^1^H NMR of JS-N-JS.

Figure 2 shows the ^1^H NMR (400 MHz, CDCl_3_) spectrum of JS-N-JS: 0.86 (t, 6H, 2CH_3_CH_2_), 1.32 (m, 56H, 2CH_3_(CH_2_)_6_CH_2_CH=CHCH_2_(CH_2_)_8_), 1.58 (s, 4H, 2CH_2_CH_2_C=O), 2.20-1.98 (m, 12H, 2CH_2_CH=CHCH_2_, 2CH_2_CH_2_NH), 2.25 (s, 4H, 2CH_2_CH_2_C=O), 2.83 (s, 1H, spacer CH_2_CH_2_NHCH_2_CH_2_), 3.37 (s, 12H, 4N^+^CH_3_), 3.42 (s, 4H, spacer CH_2_NHCH_2_), 3.7 (m, 8H, spacer CH_2_CH_2_NHCH_2_CH_2_), 4.04 (m, 4H, 2CH_2_NH), 5.41–5.30 (t, 4H, 2CH=CH), 7.93 (br, 2H, 2NH).

### 3.2. Surface Tension Measurement

**Figure 3:**
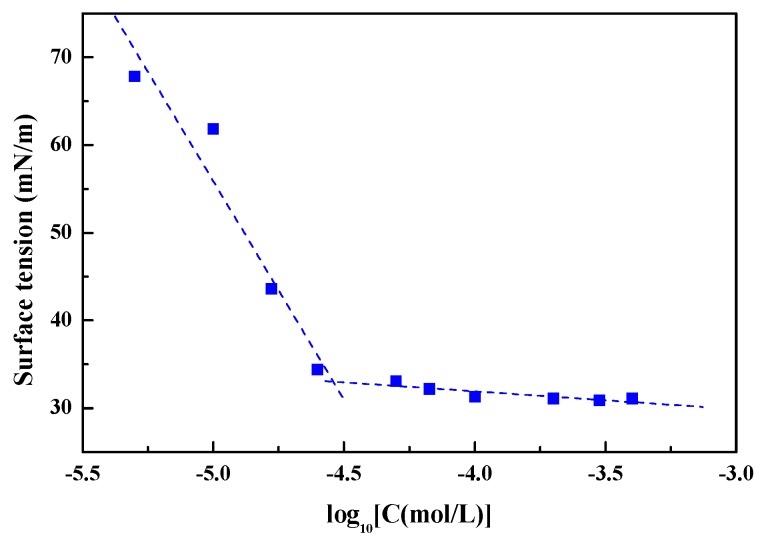
Surface tension plot for JS-N-JS surfactant.

As described before, the surface tensions of JS-N-JS solutions with different concentrations were measured by KRUSS DSA30S tensiometer at 25 °C. The measurements were conducted until the values of surface tension became stable. The plot of surface tension versus the surfactant concentrations is shown Figure 3, in which the surface tension decreased sharply with the increase of surfactant concentration at the beginning and then tended to stabilize, and the intersection point corresponds to the CMC of the JS-N-JS. Herein, the surface tension at CMC is recorded as $\gamma_{CMC}$. The CMC and $\gamma_{CMC}$ of JS-N-JS were 28.84 µmol/L and 33.12 mN/m respectively, while the other property parameters including the surface excess concentration (*Γ*_max_) and the minimum area per surfactant molecule at the aqueous solution/air interface (*A*_min_) can be calculated through Equations (1) and (2) [39]:

(1) $$\Gamma =-\frac{1}{2.303nRT}{\left(\frac{d\gamma }{d\log_{10}C}\right)}_{T,P}$$

(2) $$A_{\min}=\frac{1}{N_{A}\Gamma_{\max}}$$

where *R* is 8.314 J·mol^−1^·K^−1^, *T* is the solution temperature (K), *C* is the surfactant concentration (mol/L), the value of *n* was set to 3 in aqueous solution by investigators [41], and *N*_A_ is Avogadro’s number (6.02 × 10^23^ mol^−1^).

Compared with the Gemini surfactant C25-6-C25 and 16-E2-16, the JS-N-JS synthesized in this work exhibits superior surface activities; related parameters are listed in Table 1 [42]. The series of Gemini surfactant [43] prepared by oleylamidopropyl dimethylamine or N, N-dimethyl octadecylamine also present lower surface activities due to their shorter hydrophobic tails.

### 3.3. Conductance

**Figure 4:**
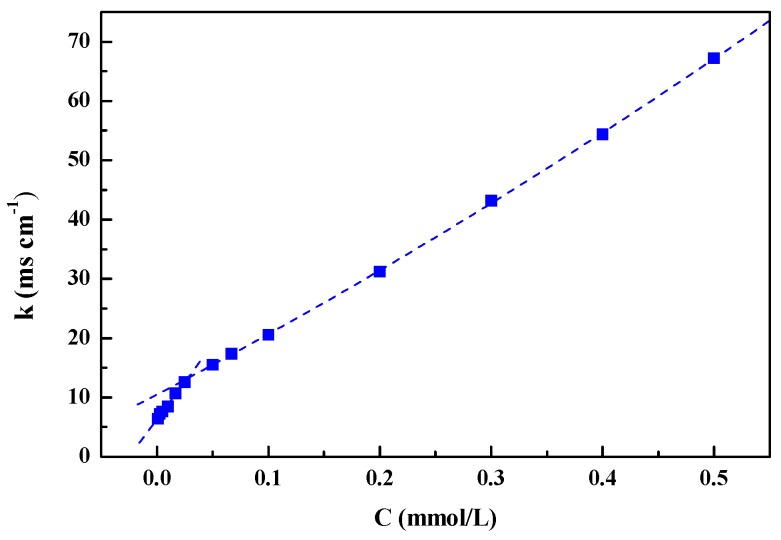
The specific conductance as a function of surfactant concentration for JS-N-JS.

The specific conductance of JS-N-JS with the variation of surfactant concentrations at 25 °C is shown in Figure 4. The intersection of the two linear segments of the *k* versus C plots in Figure 4 determined the CMC of JS-N-JS is 27.86 µmol/L, which is almost the same as that obtained by surface tension.

The ionization degree (*α*) of micelles of ionic surfactants and the binding ability of the counter-ion (*β*) can be calculated by Equations (3) and (4), respectively. The Gibbs free energy for micellization ($\Delta G_{mic}^{0}$), representing the needed energy to transfer the surfactant molecules from the monomeric form at the surface to the micellar phase, can be calculated by Equation (5). The Gibbs free energy of adsorption ($\Delta G_{ads}^{0}$), which means the energy needed to transfer 1 mol surfactant in solution to the interface, can be calculated by Equation (5).

(3) $$\alpha ={\left(\frac{dk}{dC}\right)}_{C>CMC}{\left[{\left(\frac{dk}{dC}\right)}_{C<CMC}\right]}^{-1}$$

(4) β=1−α

(5) $$\Delta G_{mic}^{0}=RT(0.5+\beta )\ln\frac{CMC}{55.4}$$

(6) $$\Delta G_{ads}^{0}=\Delta G_{mic}^{0}-\frac{\gamma_{w}\gamma_{w}-\gamma_{CMC}}{\Gamma_{\max}}$$

where ${\left(\frac{dk}{dC}\right)}_{C>CMC}$ and ${\left(\frac{dk}{dC}\right)}_{C<CMC}$ are slopes of the linear plots in Figure 4. The value of 55.4 originates that 1 L of water is equal to 55.4 mol of water at 25 °C, and the unit of CMC is mol/L. $\gamma_{w}$ is the surface tension of deionized water at 25 °C and $\gamma_{CMC}$ is the surface tension of the JS-N-JS solution at CMC. $\Gamma_{\max}$ is 1.59 µmol/m^2^, as calculated by Equation (2). The values of *α*, *β*, CMC, $\Delta G_{mic}^{0}$ and $\Delta G_{ads}^{0}$ are listed in Table 2, where these values of C25-6-C25 are used for contrastive analysis.

In Table 2, the *β* value of JS-N-JS is higher than that of C25-6-C25, while JS-N-JS and C25-6-C25 have the same molecular structure except the difference in the spacer. It was reported that the aggregation of surfactant increases with the increase of the hydrophobic tail, and the increase of charge density on the micellar surface leads to an increase of the *β* value [44]. Herein, JS-N-JS with a higher *β* value indicates that JS-N-JS is more prone to aggregate into the micellar phase than C25-6-C25, which also proves that JS-N-JS has better surface activities than C25-6-C25. The values of $\Delta G_{mic}^{0}$ and $\Delta G_{ads}^{0}$ in Table 2 are all negative, which indicates that the processes of micellization and surface adsorption are all spontaneous, no matter JS-N-JS or C25-6-C25. However, the absolute value of $\Delta G_{mic}^{0}$ of JS-N-JS is higher than that of C25-6-C25, and this also proves that JS-N-JS is more prone to aggregate into the micellar phase. Combining with the higher absolute value of $\Delta G_{ads}^{0}$ of JS-N-JS, JS-N-JS absolutely have better surface activities. The absolute value of $\Delta G_{mic}^{0}$ is always higher than that of $\Delta G_{ads}^{0}$, which indicates that the JS-N-JS molecules are more prone to be adsorbed on the surface than to aggregate into the micellar phase.

### 3.4. Salt Response

**Figure 5:**
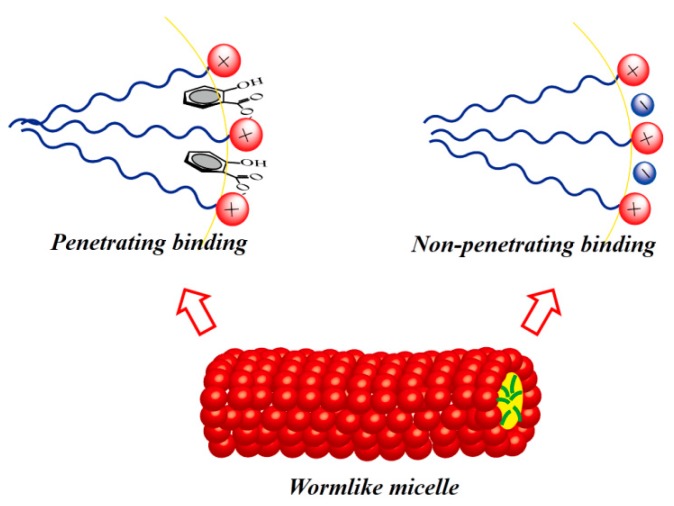
Different binding method of penetrating and non-penetrating salts: penetrating binding can increase the average volume of surfactant as well as decrease the effective head group area, while non-penetrating binding can only decrease the effective head group area.

**Figure 6:**
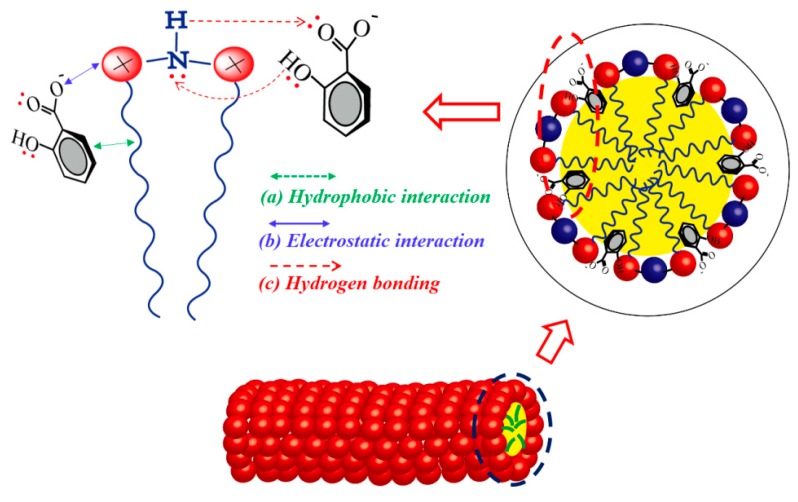
The mechanism of the formation and growing of micelle driven by sodium salicylate.

Counter-ion salts are always employed to promote the VES aggregation. In the presence of the counter-ion salt, the long-chain cationic surfactants more intend to self-assemble into micelles and undergo growth in one dimension to form long and flexible polymer-like micelles, which called wormlike micelles. Thus, the counter-ion salt is a key factor in improving the performance of VES solution, and the different counter-ions can result in different ways of packing. The counter-ion species applied in the VES micelle formation can be divided into penetrating type and non-penetrating type [45]. Salicylate is the most representative penetrating type counter-ion salt; the mechanisms of the counter-ion salts (NaSal and KCl) on VES micelle assemble is shown in Figure 5 and Figure 6. The benzene ring of salicylate penetrates the head group area and increases the average volume (*v*) of surfactant as well as the negative charge on salicylate shield the charge on the head group to decrease the repulsion and effective head group area (*a*). However, as a representative non-penetrating counter-ion, chloride ion only shields electrostatic repulsion on the surface of micelles to decrease the effective head group area (*a*). According to the packing parameter calculation formula, *P = v/a·l* [46], the penetrating type salt is more conducive to drive the growth of the micelles. Thus, the response of JS-N-JS to these two types of counter-ions needs to be investigated to achieve better performance. Herein, the influence of KCl and NaSal on the surface-active properties of the JS-N-JS solution was studied first.

**Figure 7:**
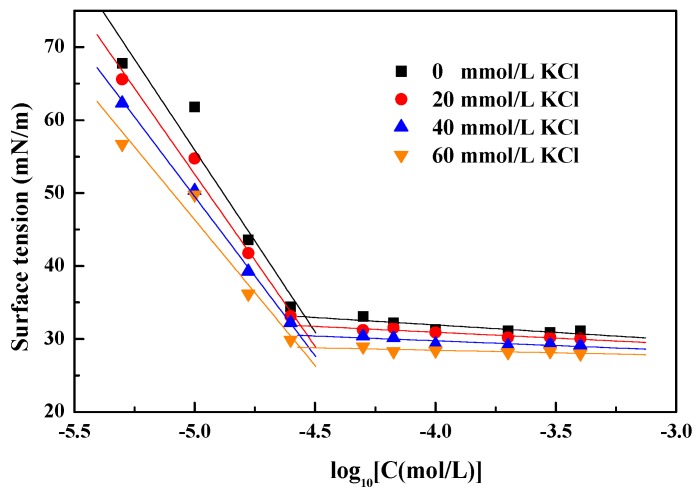
Surface tension plot for JS-N-JS surfactant at different KCl concentrations.

**Figure 8:**
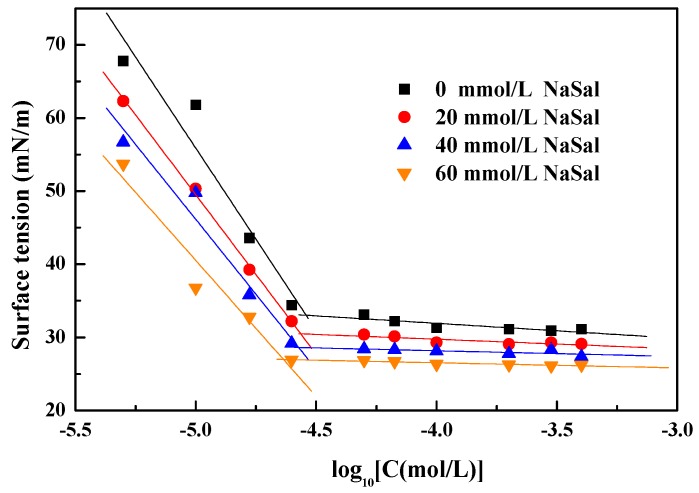
Surface tension plot for JS-N-JS surfactant at different NaSal concentrations.

The effects of salt concentration on the surface tensions of JS-N-JS solution are shown in Figure 7 and Figure 8. It is apparent that CMC decreases with the increase of the salt concentrations, and NaSal brings much more decrease than KCl. Meanwhile, Table 3 shows that the absolute value of $\Delta G_{mic}^{0}$ increases with the increase of the salt concentration. This indicates that the energy for surfactant molecule to form micelles and drive their growth was reduced by the counter-ions. Different from KCl, the effects of NaSal on the $\Delta G_{mic}^{0}$ of JS-N-JS solution can be explained in three ways, as shown in Figure 6: (i) hydrophobic interaction between the phenyl ring of NaSal and the alkyl chain of the surfactant; (ii) shielding of electrostatic repulsion; and (iii) hydrogen bonding formed between the hydroxyl on NaSal and the secondary amide on the spacer of JS-N-JS, which causes that NaSal has a more significant influence on reducing $\Delta G_{mic}^{0}$ than KCl. Thus, the JS-N-JS molecules in JS-N-JS/NaSal system are more prone to aggregate into long and flexible micelles.

**Figure 9:**
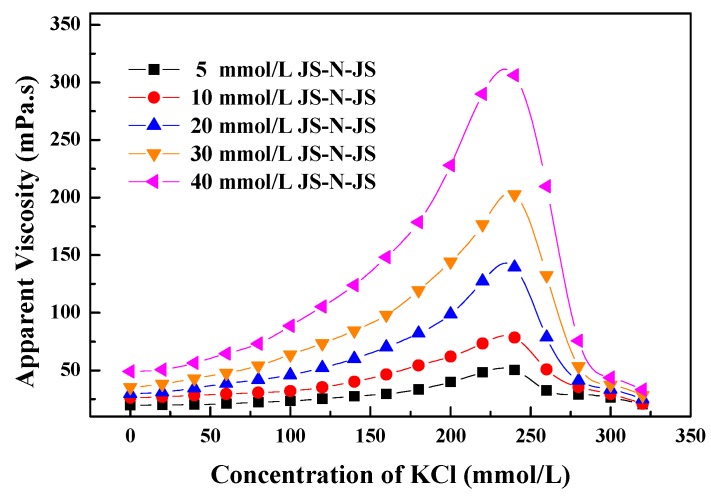
The apparent viscosity of JS-N-JS with different concentrations at different KCl concentrations.

**Figure 10:**
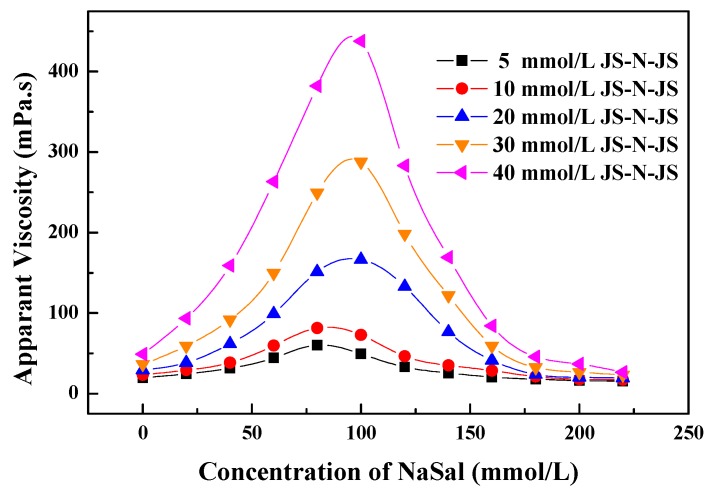
The apparent viscosity of JS-N-JS with different concentrations at different KCl concentrations.

The effect of salt concentration on apparent viscosity of VES solution was studied and the results are shown in Figure 9 and Figure 10. It is apparent that the curves of different JS-N-JS concentrations present similar trends with the increase of salt concentration. The apparent viscosity of JS-N-JS solution increases slowly with the KCl concentration ranging from 0 to 80 mmol/L and then increases significantly until the KCl concentration reaches 240 mmol/L, when the apparent viscosity peaks. With the further increase of KCl concentration, the viscosity decreases sharply. However, as shown in Figure 10, the apparent viscosity of JS-N-JS/NaSal solution increases very rapidly at the beginning, and then reaches peak value, which exhibits a significant difference to the JS-N-JS/KCl solution. It can be explained by the multi interaction between JS-N-JS and NaSal illustrated in Figure 6. The value of $\Delta G_{mic}^{0}$ in Table 3 also proves that NaSal is more conducive to the micelle aggregation and growth.

**Figure 11:**
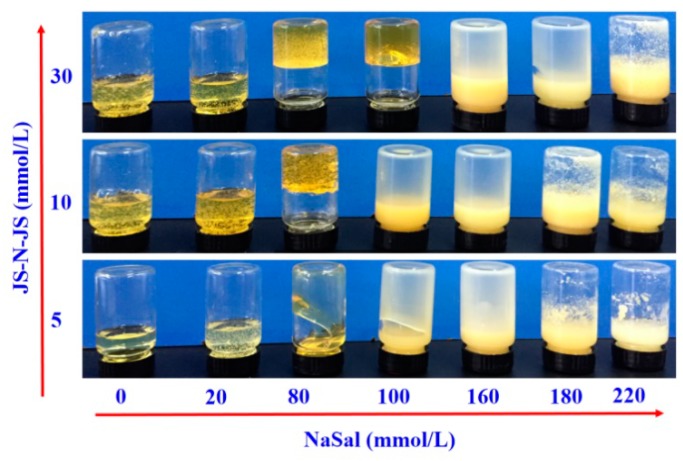
Observation of JS-N-JS aqueous solution state with an increase of NaSal concentration.

With the further increase of the counter-ion concentration after the peak viscosity, the phase separation gradually occurred in both JS-N-JS/KCl and JS-N-JS/NaSal solutions, which results in a significant drop of viscosity. The phase separation phenomenon can be observed in Figure 11. As counter-ion concentration exceeds a critical value, JS-N-JS molecules aggregate excessively, and phase separation phenomenon gradually occurred, which made the solutions become non-transparent. For the JS-N-JS/NaSal system, solutions with different JS-N-JS concentrations exhibited different critical values, as shown in Figure 10 and Figure 11. For instance, the solutions with the JS-N-JS concentrations of 5 and 10 mmol/L began to form phase separation when NaSal concentration was 80 mmol/L, while the solution with the JS-N-JS concentrations ranging from 20 to 40 mmol/L became non-transparent at NaSal concentration of 100 mmol/L. That was because fewer JS-N-JS molecules bonded with excessive NaSal and aggregated excessively into coacervation state, not wormlike micelles [47].

### 3.5. Rheological and Viscoelastic Measurement

**Figure 12:**
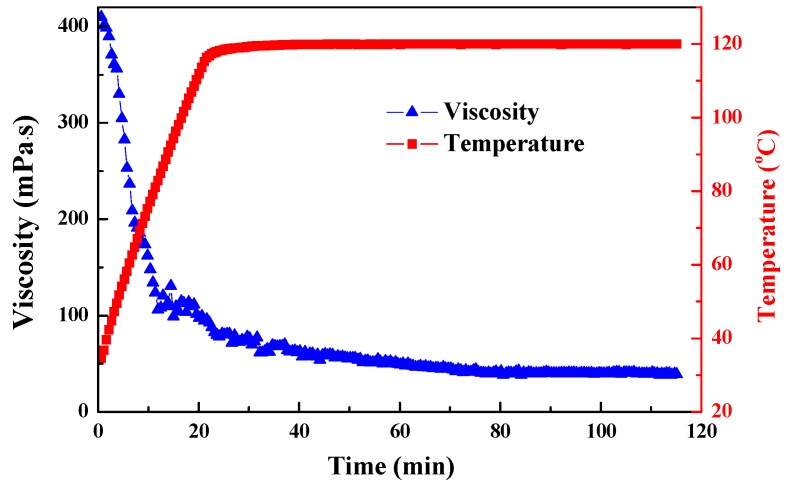
The apparent viscosity of JS-N-JS/KCl system at 120 °C and 170 s^−1^.

**Figure 13:**
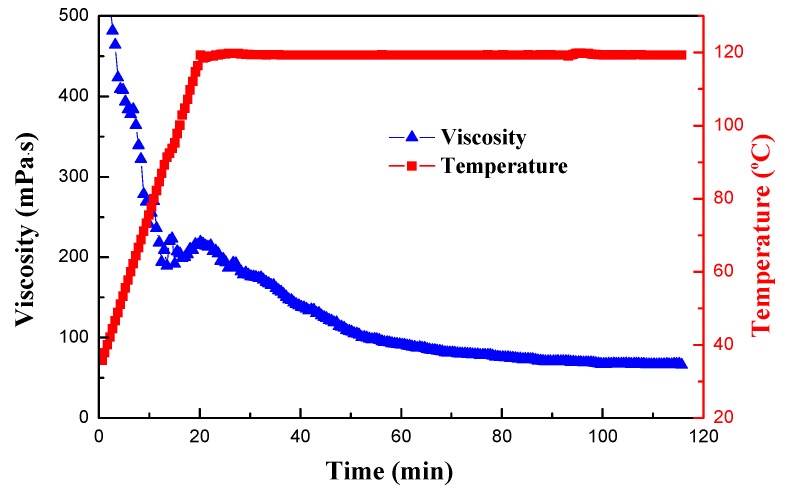
The apparent viscosity of JS-N-JS/NaSal system at 120 °C and 170 s^−1^.

The JS-N-JS/KCl and JS-N-JS/NaSal aqueous solutions exhibited a viscous state as the analysis above, both of which can be evaluated further and considered to be used as a clean fracturing fluid. Due to harsh reservoir conditions, heat and shear resistance is very crucial to fracturing fluid to ensure its sand suspending and crack creating capability. Thus, it is essential to study the temperature and continuous shearing resistance of the fracturing fluid. Since the dosage reduction of VES is an effective way to control the operation cost, the JS-N-JS concentration was set to be 30 mmol/L while the optimum concentrations of NaSal and KCl are 100 and 240 mmol/L respectively, which were applied in all performance evaluations in this work. The effect of continuous shearing and temperature on JS-N-JS/KCl and JS-N-JS/NaSal solution systems are shown in Figure 12 and Figure 13. The temperature of aqueous solution systems rose evenly from 30 to 120 °C in 20 min and the shear rate was maintained at 170 s^−1^. The apparent viscosity of JS-N-JS/KCl solution system kept at around 40 mPa·s after 110 min, while the JS-N-JS/NaSal solution system can maintain at about 70 mPa·s after 110 min. According to the SY/T 6376-2008 recommended practices for measuring the performance of water-based fracturing fluid [40], both solution systems can meet the requirement for the field application. Apparently, the JS-N-JS/NaSal solution system presented a better heat and shear resistance, which can be attributed to the multi interaction between JS-N-JS and NaSal.

Although the rheological properties are very crucial to evaluate the performance of fracturing fluid, some recent studies [48,49] suggest that fluid elasticity makes the main contribution to solid suspension capability. Thus, the viscoelastic behavior of JS-N-JS solution should be evaluated to analyze the performance of VES fracturing fluid in detail. The viscoelastic behavior of the VES solution was measured at a low angular frequency that satisfies the Maxwell model, with a single relaxation time dominating the response, which follows Equations (7) and (8) [50].

(7) $$G^{\prime }\left(\omega \right)=\frac{G_{0}\omega^{2}\tau_{R}^{2}}{1+\omega^{2}\tau_{R}^{2}}$$

(8) $$G^{\prime \prime }\left(\omega \right)=\frac{G_{0}\omega \tau_{R}}{1+\omega^{2}\tau_{R}^{2}}$$

Here, $G_{0}$ is the plateau of the storage moduli and $\tau_{R}$ is the relaxation time.

**Figure 14:**
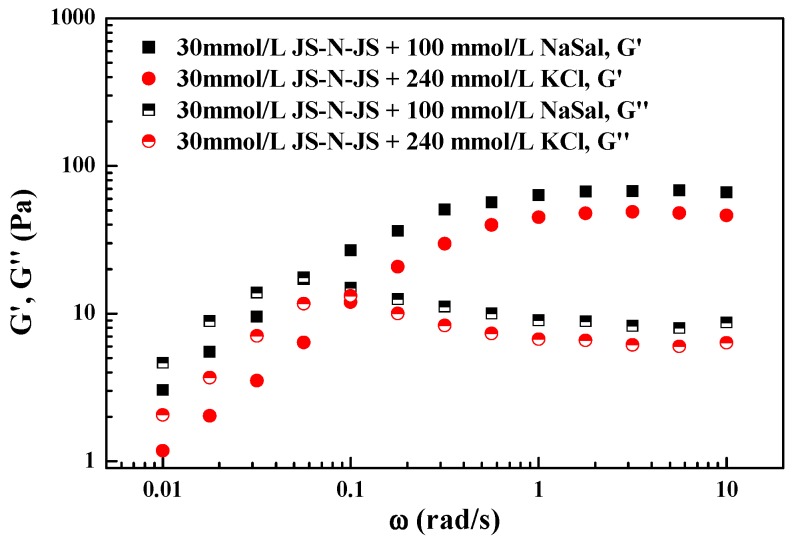
Storage moduli (G′) and loss moduli (G″) varying as a function of angular frequency.

The test results of the viscoelastic behavior of JS-N-JS aqueous solution are shown in Figure 14. It is apparent that the curves of *G′* and *G″* with a variation of oscillatory shear frequency (*ω*) of JS-N-JS/KCl and JS-N-JS/NaSal system exhibits the same trend, which shows a typical viscoelastic characteristic. At the lower oscillatory shear frequencies, the loss moduli (*G″*) is higher than the storage moduli (*G′*), and the solution behaves more viscous. As *ω* increases and exceeds the critical shear frequency (*ω_c_*), the *G′* curves cross the *G″* curves. Then, the values of *G′* are higher than *G″*, and the behavior of the solution becomes elastic. The viscoelastic property of VES solution is attributed to the network entangled by long and flexible wormlike micelles. Compared with the JS-N-JS/KCl solution, the JS-N-JS/NaSal solution has a lower *ω_c_* and indicates a higher relaxation time (*τ_R_ =* 1/*ω_c_*), and *G′* and *G″* values of JS-N-JS/NaSal solution are higher than those of JS-N-JS/KCl solution overall, which implies a stronger and tighter network of the JS-N-JS/NaSal solution.

The specific value of *G′/G″* can be used to evaluate the viscoelastic behavior: *G′/G″* < 1 means viscosity dominant, *G′/G″* > 1 means elasticity dominant, and bigger *G′/G″* means tighter network structure. Combining Equations (7) and (8), *G′/G″* follows in Equation (9) below.

(9) $$G^{\prime }/G^{\prime \prime }=\omega \tau_{R}$$

**Figure 15:**
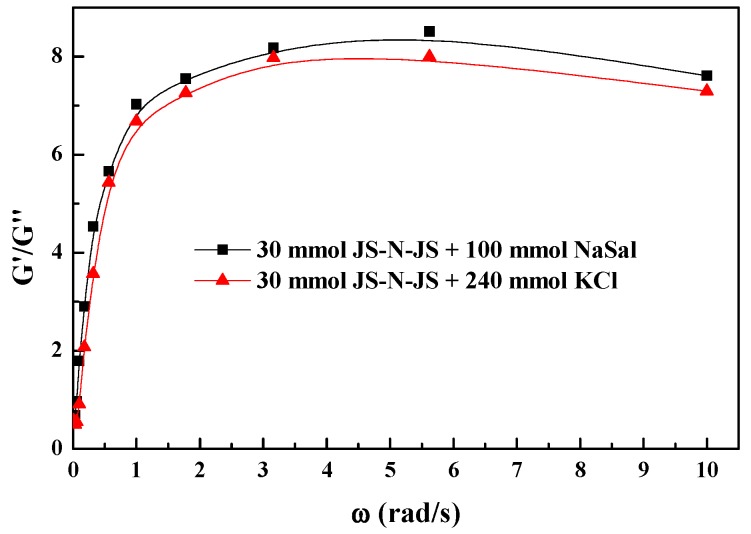
*G′/G″—ω* plots of JS-N-JS/KCl and JS-N-JS/NaSal systems.

The relationship between *G′/G″* values of the two samples and *ω* are shown in Figure 15, and the nonlinear relationship indicates that the relaxation times of the two samples are not same. By observing the variation of the curve slope, the long relaxation time existed at low frequencies while shorter at high frequencies. The *G′/G″* of the JS-N-JS/NaSal solution system is higher than that of the JS-N-JS/KCl in the whole process with the increase of the *ω*, which exhibits that the JS-N-JS/NaSal solution system owns better elastic properties and tighter network structures.

The Cole-Cole plot was employed to further investigate and compare the viscoelastic behavior of the two systems, which follows Equation (10) [51].

(10) $$G^{\prime \prime }-{\left(G^{\prime }-\frac{G_{0}}{2}\right)}^{2}={\left(\frac{G_{0}}{2}\right)}^{2}$$

**Figure 16:**
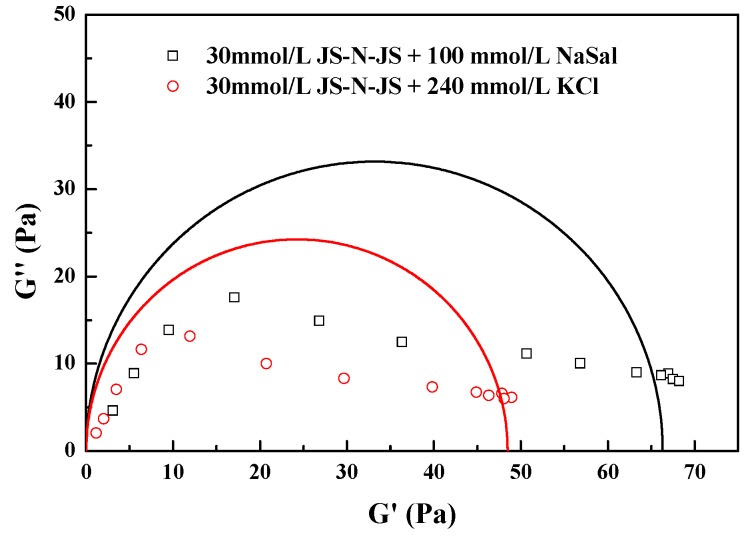
*G′/G″—ω* plots of JS-N-JS/KCl and JS-N-JS/NaSal systems.

As shown in Figure 16, the *G′*–*G″* curves of the two systems present a great deviation from the semicircular curves calculated from Equation (10) at high frequency, which is attributed to great elastic behaviors. The JS-N-JS/NaSal system presents a more significant deviation than the JS-N-JS/KCl system, which indicates a better elastic characteristic.

### 3.6. Microstructure

SEM is an efficient method to investigate the microstructure of the structural fluid directly, which can be used to prove the difference of microstructure network between JS-N-JS/NaSal system and JS-N-JS/KCl system.

**Figure 17:**
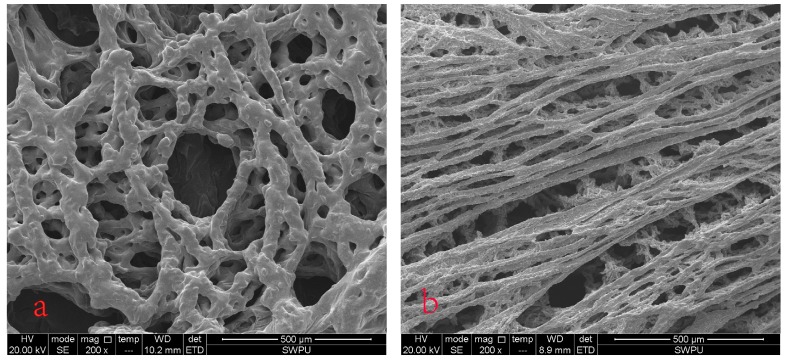
Microstructure of JS-N-JS solutions: (**a**) 30 mmol/L JS-N-JS + 100 mmol/L NaSal; and (**b**) 30 mmol/L JS-N-JS + 240 mmol/L KCl.

The microstructures of JS-N-JS solutions are shown in Figure 17, where the network of the JS-N-JS/NaSal system appears tighter, stronger and more complex than that of the JS-N-JS/KCl system. The microstructure of the JS-N-JS/NaSal system shown in Figure 17a exhibits a tough “net bag”, while the network formed in the JS-N-JS/KCl system appears as an unfinished and weak “net bag”. Therefore, the microstructure of the JS-N-JS aqueous solution also explained why the JS-N-JS/NaSal system has better heat and continuous shear resistance and elastic properties by visual proof.

### 3.7. Drag Reduction Test

Drag reduction is very important for fracturing fluid to reduce pump rate and control displacement during the fracturing operation. The VES fluid with network structure entangled by long wormlike micelles owns nice drag reduction relying on its elastic properties [13]. Due to the self-assembling property of VES molecules, wormlike micelles can recover their shapes and maintain their drag reduction ability when exposed to ultra-high shear force [14]. The drag reduction rates of the JS-N-JS/NaSal and JS-N-JS/KCl were tested and calculated by Equation (10).

(11) $$DR=(\Delta P_{w}-\Delta P_{VES})/\Delta P_{w}$$

where Δ*P_w_* is the pressure drop of purified water, and Δ*P_CMC_* is the pressure drop of JS-N-JS solution.

**Figure 18:**
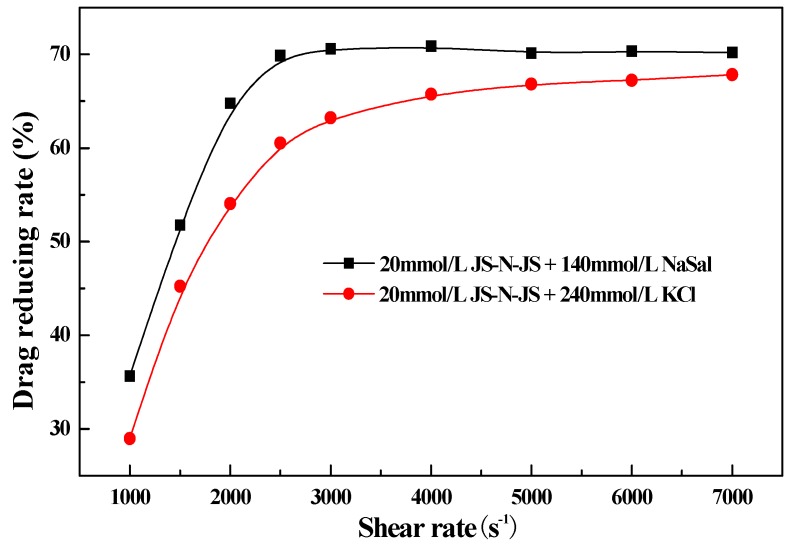
Drag reduction rate of JS-N-JS solutions at the different shear rate.

As shown in Figure 18, the JS-N-JS/KCl and JS-N-JS/NaSal systems both performs very well at an ultra-high shear rate to reduce the friction in the pipe, which is mainly attributed to their elastic properties rendered by the network microstructure. However, the drag reduction rates of the JS-N-JS/NaSal solution at each shear rate are higher than those of the JS-N-JS/KCl solution because the tighter network microstructure imparts the JS-N-JS/NaSal solution better elastic characteristic. The drag reduction rate of the JS-N-JS/NaSal solution can reach above 70% which has the same drag reduction effect as the slick-water used in a large displacement fracturing operation.

### 3.8. Proppant Suspension Measurement

Proppant-suspension capability of fracturing fluid is crucial to the success of hydraulic fracturing operation. To evaluate the proppant-suspension capability of the JS-N-JS/NaSal and JS-N-JS/KCl solution system, the static proppant suspension measurements were conducted in 50 mL measuring cylinders at 95 °C. The measurements were conducted at proppant concentrations of 30% (volume ratio) and a mesh size of 20/40. In the beginning, the proppants were evenly dispersed into the VES solution and suspended stably. Under the heating conditions, proppant settlement occurred in both systems, and the settling velocity in the JS-N-JS/NaSal system was 0.0075 mm/s, less than the 0.0103 mm/s of that in the JS-N-JS/KCl system, which was also attributed to the tighter and stronger network formed in the JS-N-JS/NaSal system. However, the settling velocity in the JS-N-JS/KCl system is less than that in C25-6-C25/KCl formula system, and one-tenth of the fracturing fluid D3F-AS05 [52]. Compared with common guar gum and polymer fracturing fluid, VES fracturing fluid exhibits better proppant suspending capability under the same viscosity due to the viscoelastic characteristic. The evaluation results show that the JS-N-JS/NaSal and JS-N-JS/KCl system both own good proppant-suspension capability, which is good for hydraulic fracturing operation.

### 3.9. Gel Breaking and Permeability Recovery Experiment

After hydraulic fracturing operation, gel breaking of fracturing fluid is an essential process to make fluids flow back easily and minimize the formation damage. The “gel breakers” used in VES fracturing fluid are external substance in the reservoir such as hydrocarbon and highly mineralized formation brines, which is different from the “internal gel breaker” added in polymer-based fracturing fluid such as ammonium persulfate and potassium persulfate. In this work, kerosene was used as an external “gel breaker” to evaluate the gel-breaking performance of the JS-N-JS solution. The JS-N-JS/NaSal solution system can be completely broken in the presence of 20% kerosene at 95 °C after 1.2 h, while the gel breaking time of the JS-N-JS/KCl solution system was 2.5 h at the same conditions. The shorter time showed that the JS-N-JS/NaSal solution system was easier to be broken, which may be attributed to that the interaction between the benzene ring and carbon chain of a hydrocarbon molecule. This disturbed the multi interaction between JS-N-JS molecule and NaSal. In the reservoir conditions, the insoluble residue may be formed due to the high temperature, which also causes damage to the reservoir. Thus, insoluble residue content and permeability recovery test were conducted at 95 °C to simulate reservoir temperature. The test results of gel breaking time, broken viscosity, insoluble residue content and permeability recovery are listed in Table 4, and all values of both systems fulfill the standards in SY/T6376-2008 to meet the requirements of the hydraulic fracturing operation.

## 4. Conclusions

In this work, a Gemini cationic surfactant of JS-N-JS with secondary amine spacer was synthesized as the VES fracturing fluid thickener and characterized by FT-IR and ^1^H NMR. The different structure of spacer imparts JS-N-JS a better performance than C25-6-C25. In the presence of KCl and NaSal, JS-N-JS molecules self-assembled into wormlike micelles which imparted the JS-N-JS solution viscoelastic properties. Due to the multi interaction between NaSal and JS-N-JS (hydrophobic interaction, electrostatic interaction and hydrogen bonding), NaSal performed better in reducing the Gibbs free energy for micellization than KCl, and the JS-N-JS/NaSal solution performed better in resistance of shearing and high temperature than the JS-N-JS/KCl solution. Combining the viscoelastic behavior test and SEM observation, it can be concluded that the network structure in the JS-N-JS/NaSal system is stronger and tighter than that of the JS-N-JS/KCl system and exhibited better elasticity. The outstanding elasticity resulted in the JS-N-JS/NaSal solution performing better in the static proppant-suspension and drag reduction tests. The fracturing fluid with the formula of 30 mmol/L JS-N-JS and 100 mmol/L NaSal can maintain a viscosity above 70 mPa·s after 110 min shearing (120 °C and 170 s^−1^). Gel-breaking and permeability recovery test results also revealed the better performance of the JS-N-JS/NaSal system. All evaluations indicated that the fracturing fluid prepared from JS-N-JS was sufficient to meet the industry standard requirements of the fracturing operation.

## Figures and Tables

**Table 1:** Surface activity properties of JS-N-JS and other contrastive surfactants.

Surfactant	*T* (°C)	CMC (µmol/L)	γ_CMC_ (mN/m)	*Γ*_max_ (µmol/m^2^)	*A*_min_ (nm^2^)
JS-N-JS	25	28.84	33.12	1.59	1.04
C25-6-C25 [39]	25	32.96	45.26	1.41	1.18
YS-YS [43]	25	196	35.2	1.18	1.41
18-18 [43]	25	191	44.7	0.99	1.73
YS-18 [43]	25	194	33.9	1.45	1.14
16-E2-16 [42]	25	1200	42.6	1.1	1.46

**Table 2:** Thermodynamics parameters.

Surfactant	*α*	*β*	CMC (µmol/L)	$\Delta G_{mic}^{0}$ (kJ/mol)	$\Delta G_{ads}^{0}$ (kJ/mol)
JS-N-JS	0.45	0.55	27.86	−36.63	−61.08
C25-6-C25	0.67	0.33	44.28	−28.88	−47.84

**Table 3:** Surface tension and CMC of JS-N-JS at different salt concentrations.

Salt (mmol/L)	CMC (µmol/L)	$\gamma_{CMC}$ (mN/m)	$\Delta G_{mic}^{0}$ (kJ/mol)	Salt (mmol/L)	CMC (µmol/L)	$\gamma_{CMC}$ (mN/m)	$\Delta G_{mic}^{0}$ (kJ/mol)
**KCl**				**NaSal**			
0	28.84	33.12	−36.63	0	28.84	33.12	−36.63
20	27.69	31.82	−37.74	20	26.87	30.78	−37.82
40	27.45	30.38	−37.77	40	25.64	28.70	−37.94
60	27.44	29.85	−37.77	60	24.85	26.73	−38.03

**Table 4:** Evaluation results of gel breaking and permeability recovery at 95 °C.

Fracturing Fluid	Gel Breaking Time (h)	Broken Viscosity (mPa·s)	Residue (mg/L)	Kerosene Permeability (mD)	Regained Permeability (mD)	Permeability Recovery Rate (%)
JS-N-JS/NaSal	1.2	2.7	46	6.49	5.52	85.05%
JS-N-JS/KCl	2.5	2.3	38	7.31	6.27	85.77%
SY/T6376-2008	≤12	≤5	≤100			≥80
